# Supplementary material for: Interventions to reduce social isolation and loneliness among minority ethnic populations in OECD countries: A scoping review
Source: PLoS One. 2024 Dec 19;19(12):e0309565. doi: 10.1371/journal.pone.0309565 (PMC11658517; doi:10.1371/journal.pone.0309565)
Supplement: S2 Table — (PDF) [file pone.0309565.s002.pdf]

S3_Table. Additional information on included studies and extraction decisions

Authors/year	country	Study title	Intervention type	Date of extraction	Name of extractor	Meets inclusion
Versey et al. (2023).	USA	A Bridging-Community (ABC) Project: A community-building social participation intervention among resettled refugees in Boston	Social facilitation	19/09/2023	EN/AA	Yes
Stewart et al. (2015)	Canada	Impacts of a support intervention for Zimbabwean and Sudanese refugee parents:" I am not alone”	Peer group mentoring	19/09/2023	EN/AA	Yes
Gater et al. (2010)	UK	Social intervention for British Pakistani women with depression: randomised controlled trial.	Social facilitation	19/09/2023	EN/AA	Yes
Stewart, et al. (2012)	Canada	Supporting African refugees in Canada: insights from a support intervention. Health & social care in the community	Social facilitation	19/09/2023	EN/AA	Yes
Lai et al. (2020)	Canada	Effectiveness of a peer-based intervention on loneliness and social isolation of older Chinese immigrants in Canada	Peer support	19/09/2023	EN/AA	Yes
Kahlon, et al (2021)	USA	Effect of Layperson-Delivered, Empathy-Focused Program of Telephone Calls on Loneliness, Depression, and Anxiety Among Adults During the COVID-19 Pandemic: A Randomized Clinical Trial	Peer support	19/09/2023	EN/AA	Yes
Kotwal, et al., (2021)	USA	A peer intervention reduces loneliness and improves social well-being in low-income older adults: A mixed-methods study	Peer support	19/09/2023	EN/AA	Yes
Lyons and Magai (2001)	USA	Reducing health risks and psychological distress among older black residents of naturally occurring retirement communities	Psychoeducational and social facilitation	19/09/2023	EN/AA	Yes
Collins and Benedict (2006)	USA	Evaluation of a community-based health promotion program for the elderly: lessons from Seniors CAN. American Journal of Health Promotion	Health promotion programme	19/09/2023	EN/AA	Yes

Hightow-Weidman et al (2015)	USA	HealthMpowerment. org: building community through a mobile-optimized, online health promotion intervention	Health education and social promotion	19/09/2023	EN/AA Conflict resolved by SP, KW	Yes
May et al (2020)	USA	The Leveraging Exercise to Age in Place (LEAP) study: engaging older adults in community-based exercise classes to impact loneliness and social isolation.	Physical and psychosocial	19/09/2023	EN/AA	Yes
Johnson et al. (2020)	USA	A community choir intervention to promote well-being among diverse older adults: Results from the community of voices trial	Physical and psychosocial	19/09/2023	EN/AA	Yes
Ehsan et al., 2021	Switzerland	Reducing loneliness in older adults: looking at locals and migrants in a Swiss case study.		26/10/2023	EN/AA Conflict resolved by SP, KW	**NO** ME participants not up to 10%
Mojica-Catillo, S (2003)	Bosnia	The effectiveness of a psychosocial group intervention on older Bosnian female refugees in diminishing loneliness		04/10/2023	EN/AA<br><br>Conflict resolved by SP, KW	**NO**<br><br>Not conducted in an OECD country)
Saito et al. 2012	Japan	Effects of a Program to prevent social isolation on loneliness, depression, and subjective Wellbeing of older adults: A randomised trial among older migrants in Japan		04/10/2023	EN/AA<br><br>Conflict resolved by SP, KW	**NO** participants not within our definition of the ME population

*ME = minority ethnicity
