# Supplementary material for: Interventions to reduce social isolation and loneliness among minority ethnic populations in OECD countries: A scoping review
Source: PLoS One. 2024 Dec 19;19(12):e0309565. doi: 10.1371/journal.pone.0309565 (PMC11658517; doi:10.1371/journal.pone.0309565)
Supplement: S1 File — (PDF) [file pone.0309565.s003.pdf]

S2. Search Strategy in CINAHL

Database: CINAHL from inception until 19th September 2023.

#	Query	Limiters/Expanders	Last Run Via	Results
S10	S3 AND S4 AND S5 AND S8	Expanders - Apply equivalent subjects<br>Narrow by Language: - English<br>Search modes - Boolean/Phrase	Interface - EBSCOhost Research Databases<br>Search Screen - Advanced Search<br>Database - CINAHL Plus with Full Text	1,533
S9	S3 AND S4 AND S5 AND S8	Expanders - Apply equivalent subjects<br>Search modes - Boolean/Phrase	Interface - EBSCOhost Research Databases<br>Search Screen - Advanced Search<br>Database - CINAHL Plus with Full Text	1,672
S8	S6 OR S7	Expanders - Apply equivalent subjects<br>Search modes - Boolean/Phrase	Interface - EBSCOhost Research Databases<br>Search Screen - Advanced Search<br>Database - CINAHL Plus with Full Text	1,140,874
S7	TI (Interventions OR programme OR program OR Campaign OR prevention OR "Preventive Health Services" OR "Health Promotion" OR "Secondary Prevention" OR "Primary Prevention" OR "Psychosocial Intervention" OR "Government Programs" OR "Tertiary Prevention" OR "Internet-Based Intervention") OR AB (Interventions OR programme OR program OR Campaign OR prevention OR "Preventive Health	Expanders - Apply equivalent subjects<br>Search modes - Boolean/Phrase	Interface - EBSCOhost Research Databases<br>Search Screen - Advanced Search<br>Database - CINAHL Plus with Full Text	1,050,198

	Services" OR "Health Promotion" OR "Secondary Prevention" OR "Primary Prevention" OR "Psychosocial Intervention" OR "Government Programs" OR "Tertiary Prevention" OR "Internet-Based Intervention")			
S6	(MH "Internet-Based Intervention") OR (MH "Program Development") OR (MH "Psychosocial Intervention") OR (MH "Government Programs") OR (MH "Community Programs") OR (MH "Program Implementation") OR (MH "Program Evaluation") OR (MH "Community Mental Health Services") OR (MH "Health Services, Indigenous") OR (MH "Health Promotion")	Expanders - Apply equivalent subjects<br>Search modes - Boolean/Phrase	Interface - EBSCOhost Research Databases<br>Search Screen - Advanced Search<br>Database - CINAHL Plus with Full Text	194,085
S5	TI ("South Asian People" OR "Southeast Asian People" OR "Black People" OR "Asian People" OR "Central Asian People" OR "india* OR pakistan* OR bangla* OR gypsy OR gypsies OR traveller* OR arab* OR asian* OR "south asia*" OR "southeast asia*" OR black OR African OR "Europe, Eastern" OR "Central America" OR "Europe" OR "South America" OR "Caribbean OR "central and eastern europe*" OR "central	Expanders - Apply equivalent subjects<br>Search modes - Boolean/Phrase	Interface - EBSCOhost Research Databases<br>Search Screen - Advanced Search<br>Database - CINAHL Plus with Full Text	214,324

europe*" OR iraq* OR Somali* OR Bosnia* OR Serbia* OR Brazil* OR "north africa*" OR "south america" OR Romanian OR roma* OR "Minority Groups" OR "Emigrants and Immigrants" OR "Ethnicity" OR "Emigration and Immigration" OR (MH "Racial Groups" OR "turkish OR albanian OR russian OR turkish OR "ethnic group*" OR "minority group*" OR Traffick* OR migrant OR refugee* OR Asylum* OR immigrant* OR immigration" OR "Ethnic and Racial Minorities" OR BAME OR BME OR “minority ethnic group” OR “ethnic minority”) OR AB ("South Asian People" OR "Southeast Asian People" OR "Black People" OR "Asian People" OR "Central Asian People" OR "india* OR pakistan* OR bangla* OR gypsy OR gypsies OR traveller* OR arab* OR asian* OR "south asia*" OR "southeast asia*" OR black OR African OR "Europe, Eastern" OR "Central America" OR "Europe" OR "South America" OR "Caribbean OR "central and eastern europe*" OR "central europe*" OR iraq* OR Somali* OR Bosnia* OR Serbia* OR Brazil* OR

	"north africa*" OR "south america" OR Romanian OR roma* OR "Minority Groups" OR "Emigrants and Immigrants" OR "Ethnicity" OR "Emigration and Immigration" OR (MH "Racial Groups" OR "turkish OR albanian OR russian OR turkish OR "ethnic group*" OR "minority group*" OR Traffick* OR migrant OR refugee* OR Asylum* OR immigrant* OR immigration" OR "Ethnic and Racial Minorities" OR BAME OR BME OR “minority ethnic group” OR “ethnic minority”)			
S4	TI (reduction or reduce or improve* or lower or decrease*) OR AB (reduction or reduce or improve* or lower or decrease*)	Expanders - Apply equivalent subjects<br>Search modes - Boolean/Phrase	Interface - EBSCOhost Research Databases<br>Search Screen - Advanced Search<br>Database - CINAHL Plus with Full Text	1,653,082
S3	S1 OR S2	Expanders - Apply equivalent subjects<br>Search modes - Boolean/Phrase	Interface - EBSCOhost Research Databases<br>Search Screen - Advanced Search<br>Database - CINAHL Plus with Full Text	209,471
S2	TI ("Social Participation" OR "Social Support" OR "Psychosocial Support Systems" OR "Social Deprivation" OR "Social Alienation" OR "Social Isolation" OR "Social Stigma" OR "Social Facilitation" OR "Social Marginalization" OR "Social Adjustment" OR "Social Cohesion" OR	Expanders - Apply equivalent subjects<br>Search modes - Boolean/Phrase	Interface - EBSCOhost Research Databases<br>Search Screen - Advanced Search<br>Database - CINAHL Plus with Full Text	57,279

	"Social Integration" OR "Social Inclusion" OR "Social exclusion" OR Lonel* OR aloneness OR Solitude) OR AB ("Social Participation" OR "Social Support" OR "Psychosocial Support Systems" OR "Social Deprivation" OR "Social Alienation" OR "Social Isolation" OR "Social Stigma" OR "Social Facilitation" OR "Social Marginalization" OR "Social Adjustment" OR "Social Cohesion" OR "Social Integration" OR "Social Inclusion" OR "Social exclusion" OR Lonel* OR aloneness OR Solitude)			
S1	(MH "Social Isolation") OR (MH "Support, Social") OR (MH "Support, Psychosocial") OR (MH "Social Alienation") OR (MH "Social Isolation (Saba CCC)") OR (MH "Social Participation") OR (MH "Social Inclusion") OR (MH "Interpersonal Relations") OR (MH "Community Programs") OR (MH "Community Role")	Expanders - Apply equivalent subjects<br>Search modes - Boolean/Phrase	Interface - EBSCOhost Research Databases<br>Search Screen - Advanced Search<br>Database - CINAHL Plus with Full Text	182,551
