# Supplementary material for: Interventions to reduce social isolation and loneliness among minority ethnic populations in OECD countries: A scoping review
Source: PLoS One. 2024 Dec 19;19(12):e0309565. doi: 10.1371/journal.pone.0309565 (PMC11658517; doi:10.1371/journal.pone.0309565)
Supplement: S2 File — (PDF) [file pone.0309565.s004.pdf]

S4. Data extraction form

General information

Study ID

Please create a study ID by using the surname of the first author, the year of publication and the initials of the publication (e.g., journal)

First author surname

Year of publication

Title

Title of paper/abstract/report that data are extracted from

Type of publication

1. Governmental report
2. Peer-reviewed journal
3. NGO report
4. Thesis
5. Other

Study funding sources

If this is not mentioned, please write NM

Possible conflicts of interest for study authors

If this is not mentioned, please write NM

Characteristics of included studies

General Methods

Aim of study

Please describe the aim of the study

Study design

1. Randomised controlled trial
2. Non-randomised experimental study
3. Cohort study
4. Cross sectional study
5. Case control study
6. Systematic review
7. Case series
8. Case report
9. Economic evaluation
10. Other

Recruitment method

Please describe the randomisation procedure (if applicable) and how participants were recruited (e.g. community setting)

Data source

Was the study based on primary or secondary data?

1. Primary
2. Secondary
3. Both

If secondary data was used, specify the database

If secondary data is used, please specify. If not, write NA (Not applicable)

Data collection method

1. Mixed method
2. Survey
3. Interviews or focus groups (please specify)

When was the data collected?

If this is not mentioned, please write NM

Study region

In which OECD country did this study take place? Please specify. If not an OECD member state write NA (not applicable):

Research question

What is the main research Question or Hypothesis (if any)

Theoretical base

What theory was the study based on? Write NM (not mentioned) if there is no evidence.

Participants

Sample characteristics

What is the target population (e.g. specific ethnicity, age, specific condition)?

1. Ethnic population
2. Age group
   Health condition
3. Sample size
4. Gender

Ethnic population
Please specify the number or percentage of participants from minority and general white populations. The minority population must be at least 10% to be included. If not mentioned, write NM

Age
If not mentioned, write NM

Health condition
If not mentioned, write NM
Sample size
If not mentioned, write NM

Gender

Write the percentage of gender participation. If not mentioned, write NM.

Inclusion criteria

If this is not mentioned, please write NM

Exclusion criteria

If this is not mentioned, please write NM

Interventions

What is the intervention description

Control

If this is not applicable, please write NA

Mode of intervention

If this is not mentioned, please write NM

Setting

If this is not mentioned, please write NM

Duration

If this is not mentioned, please write NM

Frequency

If this is not mentioned, please write NM

Delivery

If this is not mentioned, please write NM

Outcome measure

1. *Loneliness*
2. *Social isolation*

3. *Specify if other social connectedness outcomes are measured*

Tool of measurement

If this is not mentioned, please write NM

Cultural adaptation

If this is not mentioned, please write NM

Follow-up timeline

If this is not mentioned, please write NM

Main findings

What is the result?

Indicate based on the outcome measured.

Main findings

Summarise the main findings.

Conclusion

If this is not applicable, write NA

Limitation

If this is not mentioned, please write NM
