# Supplementary material for: Interventions to reduce social isolation and loneliness among minority ethnic populations in OECD countries: A scoping review
Source: PLoS One. 2024 Dec 19;19(12):e0309565. doi: 10.1371/journal.pone.0309565 (PMC11658517; doi:10.1371/journal.pone.0309565)
Supplement: Attachment — Submitted filename: Response to Reviewers.docx [file pone.0309565.s006.docx]

11^th^ July 2024

Dear Editors

**Response to Reviewers’ Comments. Re: “Dementia Risk Factors and Brain Health: A Scoping Review of Interventions to Reduce Social Isolation and Loneliness in Minority Ethnic Populations”**

We want to express our gratitude to the editors and the reviewers for their valuable feedback. We have carefully addressed all feedback below, and we believe that this has significantly strengthened the manuscript.

**Journal Requirements**

**Comments from the Editor**

Please ensure that your manuscript meets PLOS ONE's style requirements, including those for file naming

The manuscript has been revised following PLOS ONE’s style guidelines, including file naming (see pages 1, 9 and 11).

**Abstracts**

**Comments from the Editor**

Please amend either the abstract on the online submission form (via Edit Submission) or the abstract in the manuscript so that they are identical.

The abstract on the online submission form is now amended to mirror the abstract within the manuscript (see pages 1-2).

**Supporting Information file**

**Comments from the Editor**

Please include captions for your Supporting Information files at the end of your manuscript and update any in-text citations to match accordingly.

The supporting information files have now been updated with relevant captions and in-text citations (see pages 5, 7-8 for in-text citations and page 41 for captions).

**Reviewer 1: Logic and details**

I recommend a major revision to simply the scoping review of interventions in the community against SIL. If the authors absolutely insist on discussing dementia prevention, the authors should do that in the discussion section.

We have revised the manuscript to focus on those interventions that mitigate social isolation and loneliness in the community. We have changed the title of the paper to reflect this and refined the questions, the abstracts, and the introduction (see pages 1-4).

While we wanted to explore protective factors for brain health, focusing on those interventions which negate social isolation and loneliness among minority ethnic populations, we found no papers that incorporated such discussion or focus. Given the importance of social isolation and loneliness as risk factors for dementia, we have brought this element together in the discussion section (see pages 31-32).

The paper is also longer than it should be. For example, in the results section, the authors mention the scale used, however this is in the table and is not essential and should be removed.

We have removed the scale measurement in the table and reduced the introduction to ensure that we are within the necessary word count.

**Reviewer 1: Abstract**

There are repetitions, i.e., “However, despite numerous studies on reducing social isolation and loneliness in the general population, not much is known about interventions aimed at reducing these factors in minority ethnic populations as part of dementia risk reduction efforts.”

All repetitions have now been corrected and revised.

**Reviewer 1: References.**

52 and 62 are the same references. This was very confusing to the reader.

These have now been corrected with the WHO (2014) referenced at #52 and, Hynie (2018) referenced at #62.

**Reviewer 2: Title and context**

No included study was framed in the context of brain health or dementia prevention. In this long article, there is no result on dementia risk factors and brain health. This result is inevitable, however, the authors need to change the article title and Background section. (For example, short title "Dementia Risk Factors and Brain Health” is inappropriate.)

We have revised the title of this paper to exclude brain health and dementia risk factors. This now reads: Interventions to reduce social isolation and loneliness among minority ethnic populations in OECD Countries: A scoping review

I recommend the authors to exclude the context of Dementia Risk Factors and Brain Health and to review on interventions to reduce social isolation and loneliness in minority ethnic populations.

As we have previously highlighted (see Reviewer 1 logic and details), we have revised the manuscript to bring brain health into the discussion section.
