# Supplementary material for: Interventions to reduce social isolation and loneliness among minority ethnic populations in OECD countries: A scoping review
Source: PLoS One. 2024 Dec 19;19(12):e0309565. doi: 10.1371/journal.pone.0309565 (PMC11658517; doi:10.1371/journal.pone.0309565)
Supplement: Attachment — Submitted filename: Response to Reviewers.docx [file pone.0309565.s007.docx]

30^th^ July 2024

Dear Editors

**Response to Reviewers’ Comments. Re: “Interventions to reduce social isolation and loneliness among minority ethnic populations in OECD Countries: A scoping review”**

We want to express our gratitude to the editors and the reviewers for their valuable feedback. We have carefully addressed all feedback below, and we believe that this has significantly strengthened the manuscript.

**Journal Requirements**

**Comments from the Editor**

Please review your reference list to ensure that it is complete and correct. If you have cited papers that have been retracted, please include the rationale for doing so in the manuscript text, or remove these references and replace them with relevant current references. Any changes to the reference list should be mentioned in the rebuttal letter that accompanies your revised manuscript. If you need to cite a retracted article, indicate the article’s retracted status in the References list and also include a citation and full reference for the retraction notice.

The reference list has been reviewed, and incorrect references have been corrected or removed. For example,

**In Reference number 2,** WHO is now fully written as World Health Organisation.

**Reference number 34** was removed and replaced with United Nations Department of Economic and Social Affairs Population division. World Migration in Figures. OECD-UNDESA; 2013

**Reference 35** is now replaced with McAuliffe M, Triandafyllidou A. Word migration report 2022. 2021.

**Reference 69** is **now reference 66** and have been corrected from Ageing cfpo to Centre for planning on Ageing. The future ageing of the ethnic minority population of England and walesWales. 2020. Available from <http://www.cpa.org.uk/BMEprojections/BMEprojections.html>

Reference 77 is now 74 and WHO have been written in full.

**Reviewer 1: Abstract**

The finding that the interventions were divided into four categories is a very important finding, but it is not clearly mentioned in the abstract.

We have revised and included this in the abstract.

**Reviewer 1: Findings**

Also, it is unbalanced to emphasise that group activities are superior because, after all, at the current level of evidence, it is impossible to say which intervention is superior. The priority should be written in favor of the need for future research.

We have revised the manuscript to indicate that the available evidence makes it difficult to determine which intervention was superior and recommended further research. (See the abstract and page 28 of the manuscript).

**Reviewer1: Introduction**

**Also, in the second paragraph of the introduction, dementia is suddenly mentioned in detail, but it is not consistent with the whole picture. Perhaps authors do not want to delete it because it relates to the initial passion of this project, but introductions could be described in a simpler manner.**

We have revised the paragraph, identifying dementia as one amongst those other risks associated with social isolation and loneliness (see page 3 of the manuscript).

**Reviewer 1: Result**

From a medical researcher's perspective, the results section could also be shorter. It is not necessary for the reader to know detail about scale. Shorter would increase readability, but I would leave this to the editor to decide.

We have reviewed the result section and removed discussion around the different measurement scales applied across the studies, except where it formed part of the narrative of effectiveness (see pages 20-23 of the Revised manuscript with track changes or 19-23 of the Manuscript).
